# Media Discourse Regarding COVID-19 Vaccinations for Children Aged 5 to 11 Years in Australia, Canada, the United Kingdom, and the United States: Comparative Analysis Using the Narrative Policy Framework

**DOI:** 10.2196/38761

**Published:** 2024-04-29

**Authors:** Verity L Chadwick, Freya Saich, Joseph Freeman, Alexandra Martiniuk

**Affiliations:** 1 Women and Babies Ambulatory Care Royal Prince Alfred Hospital Camperdown Australia; 2 Faculty of Medicine and Health University of Sydney Camperdown Australia; 3 Dalla Lana School of Public Health University of Toronto Toronto, ON Canada; 4 The George Institute for Global Health Sydney Australia

**Keywords:** COVID-19, SARS-CoV-2, vaccine, mRNA, Pfizer-BioNTech, pediatric, children, media, news, web-based, infodemic, disinformation

## Abstract

**Background:**

Media narratives can shape public opinion and actions, influencing the uptake of pediatric COVID-19 vaccines. The COVID-19 pandemic has occurred at a time where *infodemics*, *misinformation*, and *disinformation* are present, impacting the COVID-19 response.

**Objective:**

This study aims to investigate how narratives about pediatric COVID-19 vaccines in the media of 4 English-speaking countries: the United States, Australia, Canada, and the United Kingdom.

**Methods:**

The Narrative Policy Framework was used to guide the comparative analyses of the major print and web-based news agencies’ media regarding COVID-19 vaccines for children aged 5 to 11 years. Data were sought using systematic searching on Factiva (Dow Jones) of 4 key phases of pediatric vaccine approval and rollout.

**Results:**

A total of 400 articles (n=287, 71.8% in the United States, n=40, 10% in Australia, n=60, 15% in Canada, and n=13, 3% in the United Kingdom) met the search criteria and were included. Using the Narrative Policy Framework, the following were identified in each article: hero, villain, survivor, and plot. The United States was the earliest country to vaccinate children, and other countries’ media often lauded the United States for this. Australian and Canadian media narratives about vaccines for children aged 5 to 11 years were commonly about protecting susceptible people in society, whereas the US and the UK narratives focused more on the vaccine helping children return to school. All 4 countries focused on the vaccines for children aged 5 to 11 years as being key to “ending” the pandemic. Australian and Canadian narratives frequently compared vaccine rollouts across states or provinces and bemoaned local progress in vaccine delivery compared with other countries globally. Canadian and US narratives highlighted the “infodemic” about the COVID-19 pandemic and disinformation regarding child vaccines as impeding uptake. All 4 countries—the United States, Australia, the United Kingdom, and Canada—used war imagery in reporting about COVID-19 vaccines for children. The advent of the Omicron variant demonstrated that populations were fatigued by the COVID-19 pandemic, and the media reporting increasingly blamed the unvaccinated. The UK media narrative was unique in describing vaccinating children as a distraction from adult COVID-19 vaccination efforts. The United States and Canada had narratives expressing anger about potential vaccine passports for children. In Australia, general practitioners were labelled as heroes. Finally, the Canadian narrative suggested altruistic forgoing of COVID-19 vaccine “boosters” as well as pediatric COVID-19 vaccines to benefit those in poorer nations.

**Conclusions:**

Public health emergencies require clear; compelling and accurate communication. The stories told during this pandemic are compelling because they contain the classic elements of a narrative; however, they can be reductive and inaccurate.

## Introduction

### Background

The COVID-19 pandemic has had an enormous impact on our lives, and COVID-19 vaccines remain key to preventing severe disease for most people. The media has played an important role in the pandemic, informing and educating populations about COVID-19, vaccines, and public health mitigation measures. Previous research has demonstrated that the media influences the beliefs, attitudes, and behaviors of institutions, governments, and individuals [1]. Mainstream and social media have become focal points during the pandemic, to the point where the World Health Organization (WHO) has named the excess information an “infodemic,” a term defined as “excess information including, but not limited to, false or misleading information, in digital and physical environments during an acute public health event” [2]. Disinformation and misinformation have also been features of the COVID-19 response. These are descriptions of false information, the former being deliberate to deceive and the latter not deliberate.

False and misleading information has been present for many years and has played a role in past pandemics. However, the COVID-19 pandemic has accentuated recent changes in the way we find, write, receive, and share information. Emotive misinformation travels quickly, and controversies can affect individuals, organizations, public opinion, and decision-making [3].

Infodemics and disinformation or misinformation can cause confusion and undermine the collective action needed to protect public health. There is a need to increase our understanding of the media, its messages, themes, and interpretations to improve communication in crises and ultimately improve health [4,5].

### Media

The influence of various forms of media is increasing but unpredictable. This is in part because of the opaque workings of the information delivery algorithms behind the media that is consumed digitally, as we rush to understand our rapidly changing global information landscape [6]. Current and major events in recent history have demonstrated the influence of the media on public and institutional views and actions [7]. Media can skew perceptions and cause bias [8], influence public opinion [9], and influence policy makers [10]. Notably, social media plays a major role in shaping media narratives. However, the traditional media remains an equally important influence on public opinion.

Media narratives influence people in a multitude of ways. The producers of the narrative decide which issues are salient, what suits their agenda, political leanings, and known audience, and therefore what will generate “clicks” or drive reader attention [11]. Extended coverage on issues lead us to think the issues are important and credible [12,13]. The effects of media narratives can be immediate or slow acting; can be positive or negative; and can affect individuals, institutions, and society as a whole [14].

### COVID-19 Vaccines

Vaccines are arguably the most clinically effective and cost-effective interventions in health care [15]. During pandemics, notably Zika, Ebola, Middle East Respiratory Syndrome, and now COVID-19, vaccines have been fast-tracked for approval using emergency use authorization processes for adults and subsequently for children. Emergency use authorization enables a fast track between product development and use where there is an urgent clinical need; evidence exists, but more will be gathered [16].

Public health strategies have aimed for high uptake of the COVID-19 vaccine in all age groups, as a predominantly vaccinated population will help reduce the transmission of COVID-19 and the sequelae of COVID-19 infection [1]. Achieving this goal depends on multiple aspects of the system as well as individual choice. Behavioral research identifies three categories of drivers of vaccine uptake, in addition to people having the necessary knowledge: (1) an enabling environment, (2) social influences, and (3) motivation [1]. The media plays a role in each of these, for instance, by sharing eligibility, vaccine timing, or locations where the vaccine will be offered. During the COVID-19 pandemic, many were confined to their homes or reduced their social networks, meaning perceptions of other people’s behavior—particularly regarding vaccination and also mask wearing and social distancing—were more likely to be inferred from mainstream and social media [17].

Initially, vaccinating children aged 5 to 11 years against COVID-19 was viewed as a dilemma. Although COVID-19 can be severe in adults [18], children with COVID-19 generally have a less severe disease [19]. Although only a small *proportion* of children with COVID-19 will go on to have severe disease, the widespread prevalence of COVID-19 in this population means that the absolute number is still large, including those at risk of sequential severe outcomes, particularly “long COVID” [19] or multisystem inflammatory syndrome in children [20-29]. Symptomatic or asymptomatic children may infect their parents or other susceptible people with COVID-19 [30-32]. Furthermore, children have been affected by disruptions to daycare, school, and extracurricular activities as well as the economic and mental health impacts of COVID-19 restrictions [33]. The argument for vaccinating children is strong for children with neurodisabilities; Down syndrome; immunodeficiencies; malignancies; some cardiac, respiratory, and renal diseases; obesity; and poorly controlled diabetes [34] as well as those living with high-risk household members [32]. The factors for and against vaccinating children have been described in more detail elsewhere [32].

Studies regarding the uptake of COVID-19 vaccines in children have largely focused on parents, their trust in governments, and the information they receive [35-39]. The Kaiser Family Foundation found that before approval of the vaccines for children aged 5 to 11 years in the United States, only 27% of American parents of children aged 5 to 11 years would immunize their children against COVID-19, compared with 95% of kindergarten children being immunized against several other diseases including measles, mumps, and rubella (MMR) [40]. Likewise, vaccination rates for MMR are 95% for children aged 5 years in Australia [41], 95.2% for children aged 5 years in the United Kingdom [42], and 46% to 95% for children aged 5 to 7 years [43] in Canada. In the United States, 91% of children aged 2 years and 92% of children aged 13 to 17 years are vaccinated with MMR [44]. Despite these high rates of MMR vaccination uptake, these figures are not reflected in pediatric COVID-19 vaccination rates. The latest data (July 17, 2022) in Australia for the vaccination rate for children aged 5 to 11 years is just 52.3% for first doses and 40.3% for second doses [45], despite the Pfizer-BioNTech COVID-19 vaccine for children aged 5 to 11 years being available and free since early January 2022. The disparity in vaccination rates between COVID-19 and other vaccine-preventable diseases is likely in part because of the media playing a critical role in opinions and behaviors [46].

**Figure 1:**
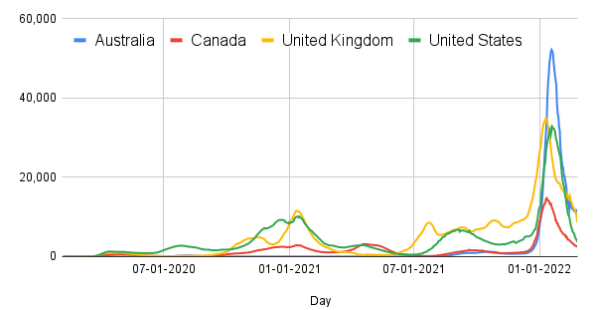
Confirmed COVID-19 cases per million during the COVID-19 pandemic.

**Figure 2:**
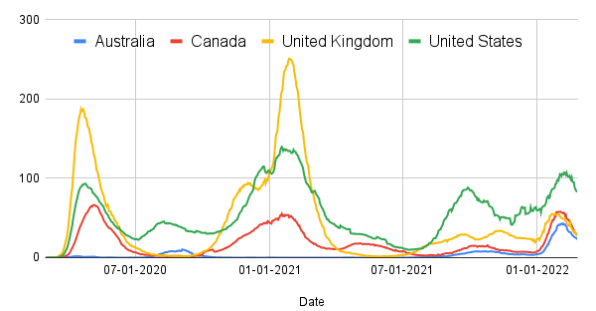
Confirmed COVID-19 deaths per million during the COVID-19 pandemic.

To date, COVID-19 and COVID-19 vaccination experiences have been vastly different in Australia, Canada, the United Kingdom, and the United States (Textbox 1). Arguably, Australia has fared the best through the pandemic, with the (until now) lowest case load (Figure 1) [47] and the lowest mortality rate (Figure 2) [47], noting that Australia has had a higher testing rate than Canada, the United Kingdom, and the United States [48]. To date (July 31, 2022), the cumulative number of cases [49] and total deaths [50] from COVID-19 infection per million people are approximately 270,971 and 3056 in the United States, 364,021 and 458 in Australia, 107,265 and 1126 in Canada, and 347,681 and 2737 in the United Kingdom, respectively. These countries have also experienced different pandemics in different health care systems. For instance, the US health expenditure per capita (US $10,921) is approximately twice that of Australia, the United Kingdom, and Canada [51]. The US health system is a complex mix of public and private, with both profit and not-for-profit insurers and providers [52]. Canada, the United Kingdom, and Australia all have universal health care provided through a government fund; however, Australia uses a mixed public or private model [53].

As COVID-19 devastated the globe, the WHO recognized the increasingly detrimental effects of the coinciding infodemic, misinformation, and disinformation on health [5]. This paper contributes new knowledge to the WHO priority of “detecting signals and understanding the spread and risk of infodemics” [2]. Given the importance of the media in shaping public and institutional knowledge, perception, attitudes, and behavior, we aimed to investigate how pediatric COVID-19 vaccine narratives have unfolded in the media of 4 countries: the United States, Australia, Canada, and the United Kingdom.

**Summary of each country’s pediatric vaccine timeline.**

**Australia**

- COVID-19 vaccines became available for children aged 5-11 years during the initial wave of Omicron, when testing capacities became overwhelmed. There were supply chain issues due too many individuals isolating after testing positive for COVID-19 or being listed as close contacts, and first child doses were due when many adults were due for their booster doses. This meant that many vaccine clinics, doctor offices, and pharmacies were inundated with the need for vaccines. Furthermore, at this time, there were supply chain issues with rapid antigen tests. This was also a holiday period for many Australians (December 2021 to January 2022), with Christmas, New Year, and school summer holidays occurring during this time.

**Canada**

- Canada approved vaccination for children aged 5-11 years shortly after the United States, and rollout started just prior to Thanksgiving (November 2021) and in time for the Christmas or New Year 2021 Holidays, around the time when the spread of Omicron was announced.

**United Kingdom**

- The United Kingdom was the last of the 4 countries to approve vaccinations for children aged 5-11 years, with the government wanting to focus on vaccinating the adult population. In January 2022, vaccines for susceptible children or children who live with susceptible adults were approved, and in late February 2022, vaccines were approved for all children aged 5-11 years, with rollout at the start of April 2022.

**United States**

- The United States was the first country to approve vaccines for children aged 5-11 years, after the pediatric society pleaded for the Food and Drugs Administration to speed up the vaccine approval for this age group, stating that the benefits outweighed the risks, and the data gathered from the ongoing trials and adult populations were sufficient. The process from approval to rollout was rapid, and the Omicron variant announcement came later.

## Methods

### Search Criteria

To better understand, compare, and contrast media narratives about the COVID-19 vaccines for children aged 5 to 11 years, we selected 4 countries. These countries were selected for specific reasons. All 4 countries are English-speaking, high-income, founding or first Organization for Economic Cooperation and Development members, democratic, and market-economy countries with multicultural populations. For each of the 4 selected countries, the federal governments have been responsible for vaccine procurement, approval, and scientific advice on their use. COVID-19 vaccines have been available for free in each of the 4 selected countries. All 4 countries had similar health expenditures per capita and, hence, were thought to be good comparators. The United Kingdom, Canada, and Australia spend very similar amounts on health per capita, whereas the United States is an outlier, spending more than twice the amount of the other listed countries per capita [51]. These 4 countries also spent similar amounts on COVID-19 in relation to their gross domestic product [54]. In Australia, Canada, and the United States, state, provincial, or territory governments have been responsible for the administration of vaccines, whereas in the United Kingdom, the deployment of vaccines is a responsibility that sits with the Department of Health and Social Care, working with the National Health Service (NHS) England and Public Health England [55]. Vaccination deployment in the rest of the United Kingdom is managed by the health services in each nation: NHS Wales, NHS Scotland, and Health and Social Care Northern Ireland [55].

We sought to better understand the narratives following advisory body statements regarding pediatric COVID-19 vaccines. Therefore, we reviewed official media releases of government advisory bodies regarding COVID-19 vaccinations for children aged 5 to 11 years published on the websites of these organizations. We then aimed to understand the mass media’s responses to these official media releases by advisory bodies.

**Figure 3:**
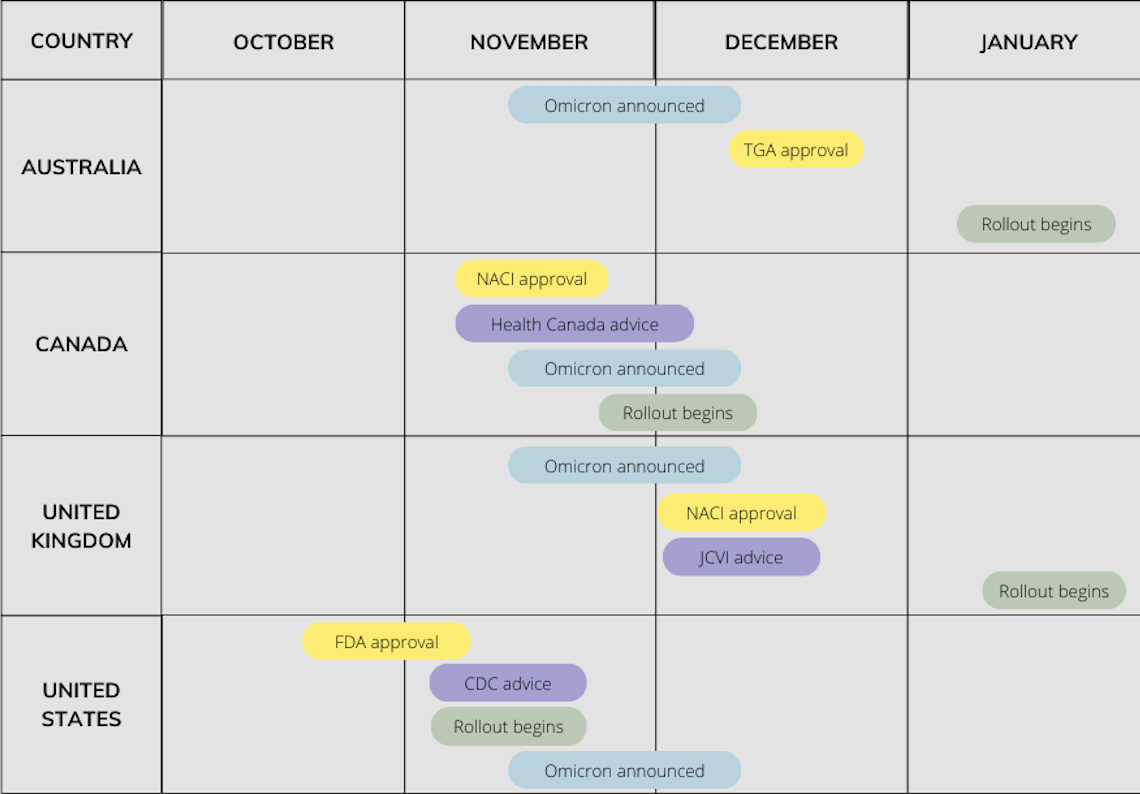
Timeline for the Pfizer-BioNTech COVID-19 vaccination in children aged 5 to 11 years in Australia, Canada, the United Kingdom, and the United States (2021 and 2022). *Only clinically susceptible children.

We searched the Factiva database, a current international news database with >30,000 news sources provided by Dow Jones, to obtain media articles for the 7-day period following 4 different announcements by advisory bodies in the 4 study countries (8 data collection segments). These announcements were (1) approval of the COVID-19 vaccines for children aged 5 to 11 years by the Food and Drug Administration (FDA), the Therapeutic Goods Administration (TGA), Health Canada, and the Medicines and Healthcare products Regulatory Agency (MHRA; United States, Australia, Canada, and the United Kingdom, respectively); (2) when vaccinating children aged 5 to 11 years was recommended or scheduled by the Centers for Disease Control and Prevention (CDC), the Australian Technical Advisory Group on Immunisation, the National Advisory Committee on Immunization (NACI), and the Joint Committee on Vaccination and Immunisation; (3) when countries started vaccinating children aged 5 to 11 years; and (4) the Omicron announcement by the WHO (November 26, 2021; Table 1; Figure 3).

We limited the searches to the top 5 media outlets in each country; refer to Multimedia Appendix 1 [56-69] for further details. The search strategy used for each of the 4 phases is detailed in Textbox 2. Duplicate articles were removed.

**Table 1:** Country-specific advisory and regulatory bodies and timeline.

Phase			Country						
			Australia		Canada		United Kingdom		United States
**Phase 1**									
	Organization	TGA^a^		NACI^b^		MHRA^c^		FDA^d^	
	Approval date	December 5, 2021		November 19, 2021		December 22, 2021		October 29, 2021	
**Phase 2**									
	Organization	ATAGI^e^		Health Canada		JCVI^f^		CDC^g^	
	Approval date	December 9, 2021		November 19, 2021		December 22, 2021^h^		December 22, 2021	
**Phase 3**									
	Date of vaccination rollout	January 10, 2022		Last week of November, 2021^i^		January 31, 2022		November 2, 2021	
**Phase 4**									
	Date of Omicron announced	November 26, 2021		November 26, 2021		November 26, 2021		November 26, 2021	

^a^TGA: Therapeutic Goods Administration.

^b^NACI: National Advisory Committee on Immunization.

^c^MHRA: Medicines and Healthcare products Regulatory Agency.

^d^FDA: Food and Drug Administration.

^e^ATAGI: Australian Technical Advisory Group on Immunisation.

^f^JCVI: Joint Committee on Vaccination and Immunisation.

^g^CDC: Centers for Disease Control and Prevention.

^h^Initially only approved for clinically susceptible children or children living with household members who are immunocompromised. On February 16, 2022, JCVI extended the offer of COVID-19 vaccination to all children aged 5 to 11 years.

^i^Varied by province.

**Factiva search strategy.**

- [Shot* OR vaccine OR immunisation OR immunization OR jab*] AND
- [child OR children OR paediatric OR pediatric OR five year OR eleven year OR 5 OR 11] AND
- [COVID OR COVID-19 OR CORONAVIRUS] AND
- United States
  [“Food and Drug Administration” OR “FDA” OR “Centers for Disease Control and Prevention” OR “CDC”]
- Australia
  [“Australian Technical Advisory Group on Immunisation” OR “ATAGI” OR “Therapeutic Goods Administration” or “TGA”]
- Canada
  [“NACI” OR “National Advisory Committee on Immunization” or “’Health Canada”]
- United Kingdom
  [MHRA OR “Medicines and Healthcare Products Regulatory Agency” OR “JCVI” OR “Joint Committee on Vaccination and Immunisation”]

### Inclusion and Exclusion Criteria

Media articles eligible for inclusion were those produced by media organization employees. To be included in further analysis, media articles were required to reference COVID-19 vaccines among children aged 5 to 11 years, the country-specific regulatory bodies, at least 1 character (hero, villain, or survivor), and a plot (in accordance with the Narrative Policy Framework [NPF] requirements); to have been published within 7 days after the event of our 4 phases detailed in Textbox 2; and to be written in English. Articles were excluded if they were based on countries other than Australia, Canada, the United Kingdom, and the United States or if they did not mention COVID-19 or vaccines for children aged 5 to 11 years.

### Analysis Framework

This study applies the NPF to compare and contrast the narratives deployed by the media as well as by advisory and regulatory bodies approving and recommending COVID-19 vaccinations for pediatric populations in Australia, Canada, the United Kingdom, and the United States and how narratives regarding the COVID-19 vaccines for children changed upon arrival of the Omicron variant. The NPF is a theoretical framework that specifies common assumptions, concepts, and hypotheses for the study of policy narratives [70] and provides guidance on how to conduct empirical research on the role of said narratives in the policy process [71]. The NPF holds that narratives have distinct components including settings (bounded by time, geography, or other characteristics), characters (heroes, villains, and survivors that may either be humans, places, or things), plots (which connect the characters to the setting), and morals (policy solutions) [46,72].

### Ethical Considerations

We did not have ethics approval for this study as no personal data was used.

## Results

### Overview

In total, 400 articles met the selection criteria (n=287, 71.8% in the United States, n=40, 10% in Australia, n=60, 15% in Canada, and n=13, 3% in the United Kingdom). The NPF was used to guide our qualitative analyses of these data to explore the framing of these media narratives about the COVID-19 mRNA vaccines for children aged 5 to 11 years. These articles were organized according to phases, which we have denoted as follows:

- Phase 1: announcement of approval by the safety governing body (FDA, TGA, NACI, and MHRA)
- Phase 2: announcement of COVID-19 vaccine approval for pediatrics aged 5 to 11 years by the country’s public health advisory body (CDC, Australian Technical Advisory Group on Immunisation, Health Canada, and Joint Committee on Vaccination and Immunisation)
- Phase 3: the rollout of vaccinating children aged 5 to 11 years
- Phase 4: the announcement of the Omicron variant (Table 1).

### Phases 1 and 2: Announcement of Approval by the Safety Governing Body (FDA, TGA, NACI, and MHRA)

There was excitement at the prospect of the COVID-19 pandemic “ending” by the US media following the CDC’s decision to approve vaccinating children, “As a mother and as a physician I know parents, caregivers, school staff and children who have been waiting for today's authorization vaccinating younger children against COVID-19, bring[ing] us closer to returning to a sense of normalcy” [73], with vaccinating children the end to worrying about doing usual activities, “it’s a total game changer for parents and families...so many parents who’ve been worried about going to work because we’re afraid of bringing back COVID to our unvaccinated children...worry about our kids going to school...surrounded by others who are not masked and may not be taking the same precautions in their homes” [74]. The US media narrative repeatedly emphasized that “vaccines will be the nail in the coffin to the pandemic” [75]. There was also repeated reassurance to parents that mandates would not be enforced, “I’m not using the M-word!,” despite acknowledging that “we already mandate vaccines for our kids, if they to go to public school” [75] because of parental concerns, “This is only emergency use. That sounds experimental to me. You’re not going to guinea pig my kid. And we’ve never really done anything like this before” [75].

In Australia, Canada, and the United States, the media accentuated the role of families protecting susceptible individuals from COVID-19 as “vaccinating children can also help reduce community transmission and help prevent children passing the virus on to younger siblings, grandparents and the wider community” [56], and the importance of vaccinating children who are susceptible to diseases, “If your child is obese, has any lung disease, heart disease, immunosuppression, this is a no-brainer. You need to get vaccinated now” [75]. In the United Kingdom, there were conflicting reports about the importance of vaccinating children to offer them protection, while at the same time considering widespread vaccination of children a “distraction” from adult vaccination efforts, “Even if the JCVI had recommended extending the age range this would not be a priority for us right now. There is a danger that if you ask the NHS to do two different things at once you lose focus and that is exactly what we don't want to happen” [76].

The US media regarding the COVID-19 vaccines for children aged 5 to 11 years emphasized the importance of attending school and being with other children with vaccines helping, “kids can go back to something that’s better than being locked at home on remote schooling, not being able to see their friends” [77], as did Canada’s media, writing that “the virus has disrupted the school year and children’s activities” [78], and the UK media, writing that “We also now have concrete evidence on the harms of children being out of school, which we must balance against the risk of harms from COVID [79],” while maintaining that “The majority of children aged five to 11 are at very low risk of serious illness due to COVID-19” [57], and therefore vaccinating and providing boosters to adults remained the priority.

During phase 1, the US media message changed from needing to get children vaccinated for herd immunity and needing to get a pediatric COVID-19 vaccination approved to end the pandemic to convincing parents that vaccination is safe and that there is “no evidence that the vaccine can lead to loss of fertility” [58]. The message to “trust your regulatory bodies” and the advice that the vaccines are safe for children was also common in Australia, “We understand parents have questions about the vaccines, especially around vaccine safety, and they need to have the opportunity to have these addressed and to access clear, trustworthy information to guide their decision-making” [59]. In particular, the initial concern and barrier to having Pfizer approved for children was “the risk of myocarditis,” but “there were no cases of myocarditis in the children’s trial,” which was key to the vaccine being approved for this age group [60]. Australian media depicted children of antivaccination parents as the survivors, “confronted with that predicament when her ex-partner started to speak out against vaccinations...Before that, the Canberra mother said she had not known her former partner to be opposed to vaccines” [61]. Similarly, in the UK media, trust in authorities was further reinforced, “Parents and carers can be reassured that no new vaccine for children would have been approved unless the expected standards of safety, quality and effectiveness have been met” [57].

US and Canadian media also focused on the winter weather, “deaths nationally have been ticking up over the past few weeks, some rural hospitals are showing signs of strain, and cold weather is setting in” [80], leading to increased COVID-19 and influenza infections, with this seen as encouragement to vaccinate children early given the expected rise in COVID-19 infections during winter.

### Phase 3: Week After Vaccine Rollout in Children

Canadian and US media stories shifted to convincing parents (with the help of quoted pediatricians) that children need to be vaccinated to help children get back to normalcy, “beyond the clinical impact...there have been detrimental social and mental health impacts that we are just beginning to fully understand. For almost two full years, school has been fundamentally changed...Tragically, COVID-19 was among the top 10 leading causes of death for children” [81]. Canada rolled out their COVID-19 vaccines for children aged 5 to 11 years a few weeks later than the United States, and the Canadian media often referred to the vaccination success of the United States, “For many Canadians, spring was a dark period marked by surging coronavirus infections, lockdowns – and the envy of watching their American neighbours get vaccinated en masse” [82]. In contrast, the Australian media narrative was predominantly expressing anger against the Morrison (federal) government for the late purchase and arrival of vaccines, and then supply chain issues causing cancelations of appointments for children’s vaccines, “My grandchildren were infected two weeks before they could get a vaccine. This need not have happened. It should not have happened. That it did is the result of the chronic incompetence of Prime Minister Scott Morrison” [62]. In the Australian media, general practitioners were painted as heroes, with media articles showing decorated offices in preparation for children’s vaccinations, but then delays to children’s vaccine appointments because “many GPs [were] unable to access adequate supplies or cope with demand, with some practices reporting receiving only 50 to 100 doses a week” [83]. Similarly, in the United Kingdom, frustration was growing as cases among children peaked owing to Omicron and children faced disruptions to schooling, “There’s a strange disconnect between the lack of mitigations and restrictions, and the lived experiences of families and schools right now” [63].

In countries with different jurisdictions, the media compared the vaccine rollouts of states or provinces. For example, in Canada, the Leader of the Opposition criticized Ontario for being too slow while British Columbia was doing much better, “provinces are taking different approaches,” with the politician noting that in “British Columbia, generally, there will not be in-school clinics during school hours” whereas “in Ontario...vaccines are being given in community clinics, hospitals and pharmacies, as well as school clinics” [64]. This was true in the United States too, with California imposing “the nation’s first coronavirus vaccine mandate for schoolchildren,” whereas “Texas Gov. Greg Abbott issued an executive order stating that no entity in Texas can mandate getting a COVID-19 vaccine,” leading to different vaccination rates across states [84]. The United Kingdom did not have the issue of discordant policies between states, but there was resistance to vaccinations taking place in primary schools, with most vaccinations taking place “at local vaccination centres or community pharmacies outside of school hours” [85]. Requiring vaccine passports was also a heated topic in the United States [86] and Canada [65], further contributing to the politicization of COVID-19.

The US media portrayed an emotive picture of brave young children lining up to get vaccinated and wanting to help the country in its fight against COVID-19, “Thousands got their first kids-size shots yesterday. And luckily they didn`t have to do it alone...comfort dogs were seen working hard to keep kids calm as they rolled up their sleeves” [87]. This de facto militarization of society against COVID-19 was echoed in Australian media, where a “command and control” military-style scale-up of the “beleaguered vaccination rollout” occurred [88]. In Canadian, UK, and US media, children were depicted as the survivors of the COVID-19 pandemic because of school closures, high case numbers, and family disruptions. Parents of susceptible children expressed relief and gratitude that their kids could get vaccinated in the United Kingdom and the United States, “On my Christmas list I said the COVID vaccine,” said Adeline Giesting, aged 11 years. “And then I was just driving in the car and I was like ‘Oh my gosh, it’s finally here! We can get the vaccine!’’’ [89].

Canadian media was less emotive than the other countries and quoted a confused public experiencing information overload, “Health Canada COVID-19 update...I was once again struck by the conflicting information being dispensed by the various authorities. There was a discussion around the intervals between vaccine doses for children; one group advocated three weeks while the other advised eight. The reason given was that each group looked at different research. Issuing these conflicting opinions only reinforces folks to do their own research and come up with their own treatment regimens, which, in many cases, does not include a vaccine” [90]. There was also reassurance provided to parents that “children tend to have robust immune responses, so reducing the dosage ensures kids will have high levels of protection while minimizing the risk of side effects” [91]. The Canadian media repeatedly suggested that parents needed to talk to their pediatrician, “Of course, pediatricians are also happy to answer questions about vaccination” [66], echoing the US media message, despite Canadians often having a family general practitioner instead of a family pediatrician.

### Phase 4: Week After the Omicron Variant Announced

Although the arrival of the Omicron variant meant that vaccines had reduced effectiveness against COVID-19 infection and against hospitalization and death for adults [92], there was less media coverage regarding the COVID-19 vaccines for children aged 5 to 11 years after Omicron arrived in comparison with the other 3 phases. In all 4 countries, Omicron (and any other new variants to come) was portrayed as the villain, posing an ongoing risk to society returning to normal. Nonetheless, reassuring messages were broadcasted, “We have more tools today to fight the variant than we’ve ever had before, from vaccines to boosters to vaccines for children, 5 years and older and much more” [93], with children wanting to go to school and families wanting to celebrate the December holidays together.

The Canadian media, and to a much lesser degree the US media, discussed low- and middle-income countries with limited vaccine availability, “When Cambodia rolled out COVID-19 vaccines, lines stretched down entire streets and people left their shoes out to save their places as they sheltered from the sun. But three months into its campaign, just 11 per cent of the population had received at least one dose” [94], compared with high-income countries with vaccine oversupply and wastage, “An informal survey shows that at least one million doses of Canada’s COVID-19 vaccine supply have gone to waste” [95]. However, the paradox in Canada was noted, “several people who have made a conscious decision on moral or ethical grounds not to get a booster, and many others...who have already foregone third doses given that large swaths of the world remain unvaccinated...[but] nobody will say, ‘I don't want to have my cancer chemotherapy because somebody in a low or middle-income country isn’t going to get their chemotherapy if I get mine’.” [67].

There was cross-country comparison by the media; in Canada, mostly with the United States; in the United Kingdom, mostly with the United States and Europe; and in Australia, mostly with the United Kingdom. Initially, the United States and Europe had higher vaccination rates in children compared with Canada, the United Kingdom, and Australia, and these countries looked to vaccinated children in the United States as real-world evidence that vaccines are safe, “Dr. Eileen de Villa, the city’s medical officer of health, told reporters ‘more than three million children have been vaccinated in the United States so far, and...there have been no major signals of concern’.” [68]. In Canada and the United States, there was substantial confusion expressed by parents regarding information overload, and in Canada, there were mixed messages about COVID-19 policies, “The vaccine promotion effort would be on considerably firmer ground, surely, if its leading lights hadn’t spent the last 20 months playing the entire country for fools - dressing up ‘operational considerations,’...as science and expecting us not to notice” (Table 2) [69].

**Table 2:** Similarities and differences in the media discourse regarding COVID-19 vaccines for children aged 5 to 11 years in 4 countries.

Phase		Australia	Canada		United States		United Kingdom			
**Similarities**										
	**Phases 1 and 2**									
		Protect susceptible individuals	—^a^		—		—			
		—	—		Children deserve to go to school, and the vaccine will help make this a possibility		—			
		Excitement regarding “ending” the pandemic with vaccinating children	—		—				—	
		—	Focus on winter weather and expected rise in COVID-19 outbreaks		—		—			
		Trust your regulatory bodies	—		—		Trust your regulatory bodies			
	**Phase 3**									
		Comparison of vaccine rollouts in different states or provinces		—		—		—		
		—	—		Parents of susceptible children expressed relief that their children can get vaccinated		—			
		—	—		Children portrayed as the survivors of COVID-19 outbreaks because of school closures		—			
		Militarization of terms regarding COVID-19	—		Militarization of terms regarding COVID-19		—			
		—	Parents encouraged to talk to their pediatrician about their concerns regarding vaccinating children		—		—			
	**Phase 4**									
		—	Discussed low- and middle-income countries with less vaccines available		—		—			
		Comparison with other countries’ more successful pediatric vaccine uptake		—		—		Comparison with other countries’ pediatric vaccine uptake		
		—	Substantial confusion because of information overload		—		—			
**Differences**										
	**Phase 1 and 2**									
		Children of antivaccination parents portrayed as the survivors		—		Children of antivaccination parents portrayed as survivors				Vaccinating children described as a distraction from adult COVID-19 vaccination efforts
	**Phase 3**									
		GPs^b^ as heroes		Referred to the pediatric vaccination success in the United States		Anger about the possibility of requiring pediatric vaccine passports				Anger of parents with ongoing schooling disruptions owing to rising COVID-19 cases
		Anger against the Morrison (federal) Government for the late purchase and arrival of vaccines and cancelation of pediatric vaccine appointments		Less emotive than other countries and expressed confusion being felt by society because of information overload		—				—
	**Phase 4**									
		—		People chose to forgo COVID-19 boosters given that many people have not yet been vaccinated globally		—				—

^a^Not available.

^b^GP: general practitioner.

## Discussion

### Principal Findings

Media narratives regarding the COVID-19 vaccine for children aged 5 to 11 years were analyzed for 4 high-income countries using the NPF. Although epidemiological models track the progression of pediatric COVID-19 infection and vaccination rates quantitatively, our analysis captures the richness of the news media portrayals and the stories behind the numbers. The media plays an important role in sharing information, shaping opinions, and ultimately influencing behavior. Analyzing media narratives offers unique clues regarding the uptake of vaccines.

The United States was the first country to vaccinate children against COVID-19, with the American Academy of Pediatrics recommending in August 2021 that the FDA speed up its approval of the Pfizer-BioNTech mRNA vaccine for children, assessing that the benefits of vaccinating this population would outweigh the risks. The primary barrier to the vaccine’s approval was concern regarding the risk of myocarditis; however, the clinical trial investigating the Pfizer-BioNTech COVID-19 vaccine in children aged 5 to 11 years found that no children developed myocarditis [60]. This led to the FDA issuing an Emergency Use Authorization for the mRNA COVID-19 vaccine for children aged 5 to 11 years, followed by Canada, Australia, and the United Kingdom approving the vaccine for this use. All countries emphasized the importance of getting children back to school and herd immunity and that vaccines would help achieve this. Initially, Australia was greatly affected by a lack of vaccine supply for adults and children, and the scarcity of vaccines was a common theme, with much blame directed at the Australian Government for its inability to deliver. However, at the same time, the Australian media (like the US media) voiced patriotism and portrayed COVID-19 as a war to win; imagery that was less prevalent in the Canadian or UK media. In contrast to patriotism, Canada had frequent media discourse regarding disadvantaged countries’ lack of access to vaccines. The United Kingdom was the last country to approve and rollout Pfizer vaccination for children, only starting to vaccinate susceptible children aged 5 to 11 years from January 30, 2022, and then all children from February 16, 2022, despite the UK medical regulator (MHRA) approving the vaccine for this age group on December 22, 2021 [96]. This decision was said to be made to avoid distracting from the adult booster vaccination efforts. Later, the media discussed the narrative of parents in the United Kingdom, particularly parents of susceptible children who were frustrated with this delay.

Television media indicated that “anti-vaxxer” frustration was rife in the United States, and similar frustration was seen in Australia through in-person demonstrations in the streets. Vaccine misinformation was (and remains) widely available. For example, the top Amazon book searches in 2021 were about how vaccines are ineffective [97], and theories on the “great reset” have gone viral [98]. However, the US mainstream print media did not report these messages in any detail. We did not find a single media article in the 4 countries studied during our study period that reported a case of severe outcome or death following the COVID-19 vaccine. We understand that reporting severe outcome or death from COVID-19 vaccines was done on social media and is thought to have had a large influence on vaccine uptake [99]. The relatively low vaccine uptake in the United States has been attributed to the subpopulations being “anti-vax” or against vaccination.

Analyses of less regulated social media (eg, TikTok and Twitter [subsequently rebranded as X]) are likely to yield different narratives. The mainstream media is built upon the premise of reporting verifiable facts and providing sources for these facts. This practice is believed to enhance the credibility of the media outlet, attracting a larger audience and consequently generating income. In contrast, social media have fewer incentives to operate in this manner and exhibit significantly less accountability [100]. According to a 2018 report, 79% of UK adults received their news from television, 64% from the internet, and 44% from social media [101]. Data from the United States in 2021 show that about half of the population receives their news from social media and the other half from traditional news outlets [102]. In Australia, a 2021 study suggests that 23% receive their news from social media [103]. In Canada, in 2021, although 52% said they read news on social media, less than one-third trusted it to be accurate [104]. Features distinct to social media, such as likes, retweets, and shares, build an ideological echo chamber with the same piece of real or fake news recirculating [105]. Other studies are exploring the role of social media narratives and their impact on COVID-19 vaccine uptake [106]. Although research into the impact of social media on COVID-19 vaccine uptake is ongoing, research into mainstream media narratives continues to be important, with the majority, or half, of the population still consuming mainstream media.

By the time Omicron was spreading globally, there was significant public fatigue about COVID-19 restrictions as well as COVID-19 information [107], and this was in part evident through significantly less media coverage of COVID-19 during this period. The media narratives of the United States and Canada also shifted to expressing increasing frustration that some people did not play their part in getting society out of this pandemic, with some saying that a key step to returning to “normal living” included the vaccination of children. The Australian media voiced this sentiment later as well, in part owing to an initial lack of Pfizer vaccine for this age group, and this sentiment was not evident in the UK media, where the vaccine for children was only recently (February 16, 2022) recommended for all children. There were some questions about whether current vaccines would protect against the Omicron variant in US media; however, this did not seem to be a theme in other countries’ narratives surrounding vaccinating children, likely because the United States authorizing the use of vaccines for children before the knowledge that Omicron was less severe and before data on the vaccines’ effectiveness against Omicron became available.

### Comparison With Prior Work

Our study contributes to the COVID-19 social science literature in the following ways: most existing COVID-19 studies on vaccinating children have focused on either the safety or efficacy of the vaccines in 5-11–year-old children or parents’ thoughts and intentions regarding vaccinating their children. This study is the first to examine the media narrative surrounding COVID-19 vaccines for children in 4 different, high-income, English-speaking countries [1]. We also dynamically tracked web-based media narratives for 4 countries following the regulatory approval of the vaccines for children aged 5 to 11 years, the rollout of vaccines, and the announcement of the Omicron variant. Given the fast-moving nature of the pandemic globally and the flurry of evolving government interventions locally, our analysis over 4 months provides a systematic picture of how media narratives in the news have shifted during the pandemic and between countries with respect to vaccination for children.

On the basis of our knowledge about media narratives, different narratives will influence the COVID-19 vaccine uptake. Pediatric vaccinations for COVID-19, as well as other pediatric vaccinations, will continue to be rolled out, and narratives in the media will continue to play an important role in vaccine uptake. The nuanced nature of the benefits of pediatric COVID-19 vaccines compared with other childhood vaccines—where the data on benefits and risks have had more time to accrue—has made the messaging of pediatric COVID-19 vaccines more challenging, and indeed, this was observed in the UK media. To date, Australian media is endorsing vaccine uptake more uniformly than US media, and the Australian pediatric uptake of the COVID-19 vaccine (adjusted for population) was more rapid than the United States’ uptake, albeit now stalled at approximately 50% [108-116].

### Limitations

This study has some limitations. First, we did not seek to analyze the media narratives of non–English-speaking countries and focused only on 4 countries. Further studies might explore media narratives present in non–English-speaking countries and vaccination rates to understand the potential influence of the media on vaccination uptake in different settings. Second, we only included studies that mentioned advisory and regulatory bodies, as we aimed to understand how the media reported on the decisions made by these bodies as well as how the media reported on professional and public opinion following these decisions. Further research could explore other themes regarding pediatric vaccines in the media. Third, we analyzed only traditional media narratives and did not explore social media narratives, which may have offered different narratives. Further research could examine social media narratives in response to advisory and regulatory bodies that approve pediatric vaccinations.

### Conclusions

Analysis of the hero, villain, survivor, and plot of 400 articles on COVID-19 vaccination in children shows that the media coverage of this pandemic relies on interpersonal narrative stories. Public health emergencies require clear; compelling; and, above all, accurate communication. The stories told during this pandemic are compelling because they contain the classic elements of a narrative; however, they can be reductive and inaccurate. The media analysis presented in our paper is significant. It assists in informing how health policy and guidelines are presented to the public in the mainstream media, particularly as the science behind those policies and guidelines is unfolding in real time. Understanding the role of the mainstream media in health communications will remain crucial as we continue through the COVID-19 pandemic as well as for future health crises.

## Appendix

Multimedia Appendix 1 Sources for top 5 media outlets per country.

## Abbreviations

CDC: Centers for Disease Control and Prevention
FDA: Food and Drug Administration
MHRA: Medicines and Healthcare products Regulatory Agency
MMR: measles, mumps, and rubella
NACI: National Advisory Committees on Immunization
NHS: National Health Service
NPF: Narrative Policy Framework
TGA: Therapeutic Goods Administration
WHO: World Health Organization
