# Supplementary material for: Media Discourse Regarding COVID-19 Vaccinations for Children Aged 5 to 11 Years in Australia, Canada, the United Kingdom, and the United States: Comparative Analysis Using the Narrative Policy Framework
Source: JMIR Form Res. 2024 Apr 29;8:e38761. doi: 10.2196/38761 (PMC11060323; doi:10.2196/38761)
Supplement: Multimedia Appendix 1 [file formative_v8i1e38761_app1.docx]

**Appendix 1: Article samples from top media outlets by country**

| **Country** | **Top media outlets** | **Source** |
| --- | --- | --- |
| **Australia** | ABC News, 9news, the Sydney Morning Herald, The Guardian | 56,59,61,62,63 |
| **Canada** | The Globe and Mail, The Toronto Star, National Post, Toronto Sun, Financial Post | 64,65,66,67,68,69 |
| **UK** | The Sun, The Sun on Sunday, Daily Mail, Mail on Sunday, Metro | 57 |
| **USA** | CNN, MSN, Fox news, New York Times, Finance Yahoo | 58,60 |
